# Recombinant *Mycobacterium smegmatis* with a pMyong2 vector expressing Human Immunodeficiency Virus Type I Gag can induce enhanced virus-specific immune responses

**DOI:** 10.1038/srep44776

**Published:** 2017-03-16

**Authors:** Byoung-Jun Kim, Jeong-Ryeol Gong, Ga-Na Kim, Bo-Ram Kim, So-Young Lee, Yoon-Hoh Kook, Bum-Joon Kim

**Affiliations:** 1 Department of Microbiology and Immunology, Biomedical Sciences, Liver Research Institute and Cancer Research Institute, College of Medicine, Seoul National University, Seoul, Korea

## Abstract

Recently, we have developed a novel *Mycobacterium*-*Escherichia coli* shuttle vector system using pMyong2, which can provide an enhanced expression of heterologous genes in recombinant *Mycobacterium smegmatis* (rSmeg). To investigate the usefulness of rSmeg using pMyong2 in vaccine application, we vaccinated *M. smegmatis* with pMyong2 system expressing Human Immunodeficiency Virus Type I (HIV-1) Gag p24 antigen (rSmeg-pMyong2-p24) into mice and examined its cellular and humoral immune responses against HIV gag protein. We found that rSmeg-pMyong2-p24 expressed higher levels of Gag protein in bacteria, macrophage cell line (J774A.1) and mouse bone marrow derived dendritic cells (BMDCs) compared to rSmeg strains using two other vector systems, pAL5000 derived vector (rSmeg-pAL-p24) and the integrative plasmid, pMV306 (rSmeg-pMV306-p24). Inoculation of mice with rSmeg-pMyong2-p24 elicited more effective immunity compared to the other two rSmeg strains, as evidenced by higher levels of HIV-1 Gag-specific CD4 and CD8 T lymphocyte proliferation, interferon gamma ELISPOT cell induction, and antibody production. Furthermore, rSmeg-pMyong2-p24 showed a higher level of cytotoxic T cell response against target cells expressing Gag p24 proteins. Our data suggest that *Mycobacterium*-*Escherichia coli* shuttle vector system with pMyong2 may provide an advantage in vaccine application of rSmeg over other vector systems.

An effective human immunodeficiency virus (HIV) vaccine will likely need to elicit virus-specific neutralizing antibodies and cytotoxic T-lymphocyte (CTL) responses. Although an immunogen that induces antibodies that can neutralize diverse HIV type 1 (HIV-1) isolates has not yet been defined, a number of strategies including plasmid DNA vaccine, adenovirus serotype 5, and pox-vectored vaccine are being developed for generating HIV-1-specific CTL^1^. However, there are problems associated with each of these approaches with regard to eliciting CTL, which could limit their practical uses.

Mycobacteria have features that make them attractive as potential HIV-1 vaccine vectors. *Mycobacterium bovis* BCG (BCG), currently the most widely administered vaccine in the world, is a live attenuated vaccine used to protect against tuberculosis and leprosy^2^,^3^,^4^,^5^,^6^. It demonstrates excellent adjuvant properties, induces long lasting immunity and has a low production cost^7^,^8^,^9^. It also has many properties that make it one of the most attractive live vectors for the development of recombinant vaccines in murine models against various infectious agents, including *Borrelia burgdorferi, Streptococcus pneumoniae, Bordetella pertussis*, rodent malaria, leishmania, and measles virus^10^,^11^,^12^,^13^,^14^,^15^. *Mycobacterium smegmatis*, a non-pathogenic member of the genus *Mycobacterium*, grows rapidly and can be transformed effectively with many genes *in vitro*^16^. These properties make this bacterium an ideal vaccine vector^17^,^18^,^19^,^20^. It has been reported that recombinant *M. smegmatis* (rSmeg) engineered to express HIV-1 Env elicits HIV-1 envelope-specific CD8 T-cell responses^17^. Unlike other mycobacterial species, such as BCG that can survive in host cells by inhibiting phagosome maturation, *M. smegmatis* is rapidly destroyed by phagolysosomal proteases in the phagosomes of infected cells^21^,^22^, facilitating the rapid uptake of expressed antigens in bacteria and cross-presentation of antigen into T cells. Furthermore, *M. smegmatis* can induce cytokine production by macrophages better than pathogenic mycobacterial species and can activate and induce the maturation of dendritic cells better than BCG by the upregulation of major histocompatibility complex (MHC) class I and costimulatory molecules^23^,^24^,^25^. *M. smegmatis* can also access the MHC class I pathway for presentation of mycobacterial antigens more efficiently than BCG, suggesting it has an advantage in inducing CTL response, which is necessary in HIV vaccine^26^,^27^.

Despite the intrinsic trait of mycobacteria in inducing CTL response, there is one pitfall in the application of recombinant mycobacteria into vaccine application, which is the lack of stability and the levels of heterologous expression of a foreign gene. Thus, there is an urgent need for the development of a novel *Mycobacterium*-*Escherichia coli* shuttle vector system which can improve upon conventional systems. Recently, we have introduced a novel *Mycobacterium*-*Escherichia coli* shuttle vector system using pMyong2, a linear plasmid of the slowly growing *Mycobacterium yongonense* DSM 45126^T^ strain. Of note, we found that rSmeg with the pMyong2 vector system increased the a copy number of human macrophage migration inhibitory factor (hMIF) gene approximately 37-fold and increased the protein expression of hMIF approximately 50-fold compared to rSmeg with the pAL5000 vector system, the most widely used vector for heterologous expression of foreign genes in mycobacteria, demonstrating the potential utility of the pMyong2 vector system in heterologous gene expression in rSmeg^28^.

The aim of the present study is to investigate the usefulness of rSmeg with pMyong2 in HIV vaccine application. To this end, we constructed the rSmeg with pMyong2 system expressing HIV-1 p24 Gag antigen (rSmeg-pMyong2-p24) and examined its cellular and humoral immune responses against HIV Gag proteins in vaccinated mice compared with rSmeg strains transfected with 2 other vector systems, an episomal plasmid, pAL5000 derived vector (rSmeg-pAL-p24) and an integrative plasmid, pMV306 (rSmeg-pMV306-p24).

## Results

### The rSmeg-pMyong2-p24 strain elicited enhanced HIV-1 p24 Gag expression in bacteria and in an infected murine macrophage cell line

**Figure 1:**
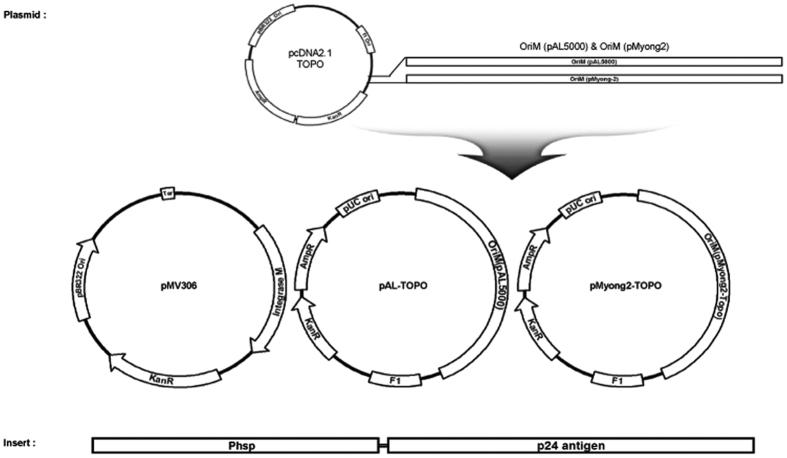
Maps of p24 expression vectors of the study. Maps of the constructed p24 expression *Mycobacterium*-*E. coli* shuttle vectors. pMV306-p24, pAL-p24 and pMyong2-p24 vectors expressed p24 under control of the *hsp65* promoter from *M. bovis* BCG.

**Figure 2:**
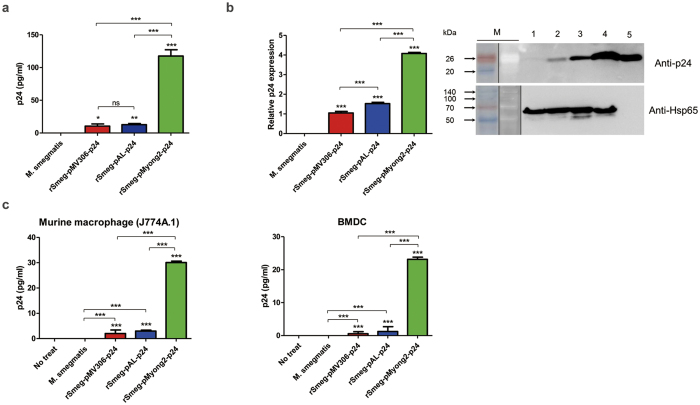
Levels of p24 expression by rSmeg strains and murine macrophages infected with rSmeg strains. (**a**) Confirmation of p24 expression in recombinant *M. smegmatis* by ELISA. (**b**) Confirmation of p24 expression in recombinant *M. smegmatis* by Western blot. Proteins were extracted from wild-type *M. smegmatis* (lane 1) and rSmeg strains (lane 2, rSmeg-pMV306-p24; lane 3, rSmeg-pAL-p24; lane 4, rSmeg-pMyong2-p24). Purified p24 protein was used as a positive control (lane 5). M, molecular weight standard (Elpis Bio, Taejeon, Korea; DokDo-MARK^TM^). Distinct membranes were separated by white space. And, marker lane was separated by vertical black line. The expression levels of p24 are plotted. The Western blot image was cropped from a full-length blot for improving the clarity and conciseness. The full-length blot image is presented in Supplementary Fig. S6. (**c**) Levels of p24 expression after infection of the murine macrophage cell line J774A.1 (left panel) and BMDCs (right panel) with rSmeg strains (lane 2, rSmeg-pMV306-p24; lane 3, rSmeg-pAL-p24; lane 4, rSmeg-pMyong2-p24).

To explore the usefulness of pMyong2 vector system in the generation of rSmeg strains for HIV-1 p24 Gag vaccination, we generated a total of 3 types of rSmeg strains expressing p24, rSmeg-pMyong2-p24, rSmeg-pAL-p24 and rSmeg-pMV306-p24 using different types of *Mycobacterium*-*E. coli* shuttle vectors, pMyong2-TOPO^28^, pAL-TOPO^28^, and pMV306^29^, respectively (Fig. 1). When the growth rates of the 3 rSmeg strains in 7H9 broth (with 100 μg/ml of kanamycin) for 5 days were compared with each other, rSmeg-pMyong2-p24 strains showed retardation in growth in the interval from 6 hrs to 48 hrs. But, after 48 hrs, all the 3 strains showed almost the same growth rate (Supplementary Fig. S1). To compare the levels of p24 expression in bacteria among the 3 rSmeg strains, we conducted ELISA analysis (Fig. 2a) and Western blot (Fig. 2b) against p24 after lysis of cultured bacteria. All the rSmeg strains could express the p24 protein. But, differences in p24 expression levels were found among the 3 strains, particularly between rSmeg-pMyong2-p24 and the other 2 strains. The rSmeg-pMyong2-p24 produced approximately five to ten times higher levels of p24 than other strains. The rSmeg-pAL-p24 produced slightly higher level of p24 than rSmeg-pMV306-p24 (Fig. 2a and b). To check the stable expression of p24, the p24 expression levels from the rSmeg-pMyong2-p24 strain of various passage points were also determined on 7H10 agar plates with or without kanamycin. In strains passaged on the 7H10 agar plates with kanamycin, even after 12^th^ passages, the rSmeg-pMyong2-p24 strain could express stable p24 antigen (Supplementary Fig. S2). However, in strains passaged on the 7H10 agar plate without kanamycin, its stability of p24 expression was sharply decreased after 6^th^ passages (Supplementary Fig. S3), due to the loss of pMyong2-TOPO vector of high copy number capable of inducing overexpression of exogenous p24 antigens. To determine whether the difference in p24 expression among the 3 strains could be recapitulated in infected macrophages and infected BMDCs, we checked the p24 level by ELISA in infected J774A.1 cells and mouse BMDCs. Generally, similar trends noted in lysed bacteria were also observed in infected cells, but the differences were more pronounced (Fig. 2c). Taken together, our data indicated that rSmeg-pMyong2-p24 showed an increased production of p24 in infected macrophages as well as in bacteria, compared to the other two rSmeg strains, rSmeg-pAL-p24 and rSmeg-pMV306-p24. This finding suggests that the former has the potentials to elicit an enhanced p24 antigen specific immune response in vaccinated animals or humans by loading more p24 antigen into antigen presenting cells, compared to the latter two strains.

### BMDC infected with rSmeg-pMyong2-p24 strain elicited enhanced T cell proliferation from mouse T cell immunized by HIV-1 p24 Gag

**Figure 3:**
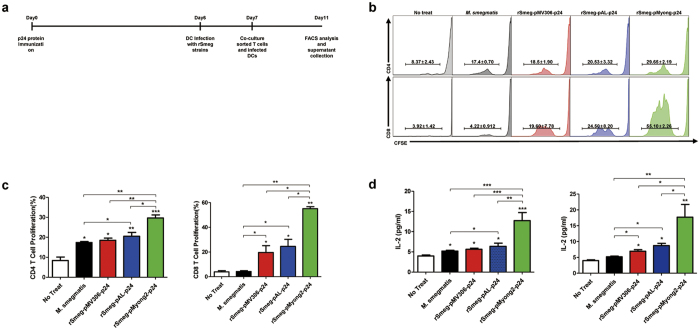
Levels of T cell proliferation induced by BMDCs infected with p24 recombinant *M. smegmatis* strains. (**a**) Schematic schedule for the T cell proliferation assay. (**b** and **c**) Flow cytometric analysis of the proliferation of CFSE-labeled CD4 and CD8 T cells due to BMDCs infected with p24 recombinant *M. smegmatis* strains. (**d**) ELISA determination of IL-2 released in the supernatants of CD4 (left panel) and CD8 (right panel) cells in a MLR assay. Data are representative of 3 independent experiments. All the statistical analyses were calculated by comparisons with the values for rSmeg-pMyong2-p24. Means ± SD are shown. *P < 0.05; **P < 0.01; ***P < 0.001.

To test whether rSmeg-pMyong2-p24 enhanced p24 protein production has improved T cell proliferation capacity, we conducted a T cell proliferation assay of infected BMDCs with 3 different types of rSmeg strains, rSmeg-pMyong2-p24, rSmeg-pAL-p24 and rSmeg-pMV306-p24 and a wild-type strain as a control measuring CFSE dye dilution in a mixed lymphocyte reaction (MLR) assay^30^. The schematic schedule for T cell proliferation assay is described in Fig. 3a. All the BMDCs infected with the 4 Smeg strains (3 rSmeg and 1 wild-type) induced significantly higher levels of CD4 T cell proliferation, compared to BMDCs not infected with *M. smegmatis* strains. Of note, BMDCs infected with rSmeg-pMyong2-p24 induced significantly higher levels of CD4 and CD8 T cell proliferation compared to the other two rSmeg strains as well as the wild-type strain. No significant difference between the rSmeg strains rSmeg-pAL-p24 and rSmeg-pMV306-p24 could be found. Even in CD4 T cell proliferation, BMDCs infected with rSmeg-pAL-p24 or rSmeg-pMV306-p24 showed similar proliferation levels to that of the wild- type (Fig. 3b and c). Comparison of IL-2 amounts from stimulated CD4 and CD8 T cells also showed similar trends as shown in T cell proliferation assays. The result showed that BMDCs infected with rSmeg-pMyong2-p24 induced significantly higher levels of secreted IL-2 from both CD4 and CD8 T cells, compared to BMDCs infected with the other 2 rSmeg strains and wild-type strain (Fig. 3d). Taken together, our data indicated that rSmeg-pMyong2-p24 induced increased T cell proliferation, maybe due to enhanced antigen loading into antigen presenting cells such as DCs, resulting in potentiating vaccine efficacy.

### The rSmeg-pMyong2-p24 strain elicited enhanced HIV-1 p24 Gag-specific IFN-γ spot forming cells (SFC) in mouse spleens generated by subcutaneous immunization

**Figure 4:**
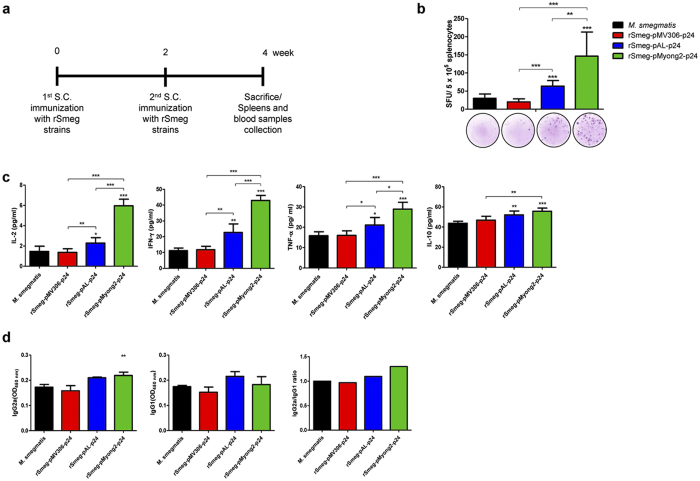
*In vivo* immune responses induced by p24 recombinant *M. smegmatis* strains. (**a**) Schematic immunization schedule for *in vivo* immunological assays. (**b**) Levels of IFN-γ secretion levels by *in vitro* stimulated splenocytes from vaccinated mice with p24 recombinant *M. smegmatis* strains were detected with ELISPOT analysis. Representative images of ELISPOT membrane in each group are shown below the graph. (**c**) Levels of IL-2, IFN-γ, TNF-α, and IL-10 cytokines by *in vitro* stimulated splenocytes with p24 from mice vaccinated with p24 recombinant *M. smegmatis* strains were detected with ELISA analysis. For the detection of IL-2 and TNF-α cytokines, splenocytes were stimulated with p24 for 24 hr; for IFN-γ and IL-20, splenocytes were incubated with p24 for 72 hr. (**d**) p24 specific immunoglobulin subtypes (IgG2a and IgG1) were detected by ELISA at 450 nm. OD values for IgG2a and IgG1 subtypes and the ratio of IgG2a/IgG1 were compared. All the statistical analyses were calculated by comparisons with the values for rSmeg-pMyong2-p24. Means ± SD are shown. *P < 0.05; **P < 0.01; ***P < 0.001.

To test whether rSmeg-pMyong2-p24 has improved T cell response after vaccination, splenocytes were isolated from spleens of BALB/c mice subcutaneously (s.c.) immunized with 3 different types of rSmeg strains, rSmeg-pMyong2-p24, rSmeg-pAL-p24 and rSmeg-pMV306-p24 (~10^6^ CFU) (Fig. 4a) and a wild- type strain as a control and assayed for HIV-1 p24 Gag- specific T cell responses using IFN-γ ELISPOT assays. Splenocytes from s.c. immunized mice with two rSmeg strains, rSmeg-pMyong2-p24, and rSmeg-pAL-p24 yielded significantly higher SFUs than those of wild-type and rSmeg-pMV306-p24 strains. Of note, splenocytes from mice immunized with rSmeg-pMyong2-p24 (146.33 ± 66.91 SFUs/5 × 10^5^ splenocytes) yielded significantly higher SFUs than those of the 2 other rSmeg strains, rSmeg-pAL-p24 (63.63 ± 15.72 SFUs/5 × 10^5^ splenocytes) and rSmeg-pMV306-p24 (63.63 ± 15.72 SFUs/5 × 10^5^ splenocytes) (Fig. 4b). Interestingly, a significant difference in SFC numbers was also observed between the two rSmeg strains rSmeg-pAL-p24 and rSmeg-pMV306-p24, which showed no or little difference in T cell proliferation or p24 expression. Taken together, our data indicated that rSmeg-pMyong2-p24 elicited an improved effector T cell function in vaccinated animals.

### The rSmeg-pMyong2-p24 strain produces cytokines related with Th1 immune response

Splenocytes obtained two weeks after the second immunization with rSmeg strains (Fig. 4a) were stimulated *in vitro* with purified p24 protein (5 μg/ml), and the induced production of IL-2, IFN-γ, TNF-α and IL-10 cytokines were measured in the cell culture supernatants. In the case of rSmeg-pMyong2-p24 immunized splenocytes, the levels of all the Th1 immune response related cytokines (IL-2 for rSmeg-pMV306-p24 vs. rSmeg-pAL-p24 vs. rSmeg-pMyong2-p24: 1.37 ± 0.37 vs. 2.27 ± 0.54 vs. 5.95 ± 0.66 pg/ml; IFN-γ: 11.78 ± 2.16 vs. 22.67 ± 5.44 vs. 42.95 ± 3.21 pg/ml and TNF-α: 16.00 ± 2.26 vs. 21.08 ± 3.77 vs. 28.92 ± 3.41 pg/ml, respectively) were significantly higher than those of wild- type or rSmeg strains. The rSmeg-pMyong2-p24 strain also increased the level of IL-10 release; however, all the strains showed similar levels (rSmeg-pMV306-p24 vs. rSmeg-pAL-p24 vs. rSmeg-pMyong2-p24: 43.88 ± 3.87 vs. 52.12 ± 3.85 vs. 55.72 ± 3.12 pg/ml) (Fig. 4c).

### The rSmeg-pMyong2-p24 strain elicits a HIV-1 p24 Gag-specific Th1-biased humoral response in immunized mice

To test whether rSmeg-pMyong2-p24 elicits a Th1-biased humoral response in immunized mice, we analyzed the levels of HIV-1 p24 Gag-specific IgG2a and IgG1, which are known as markers for Th1 and Th2 responses, respectively^31^,^32^,^33^. The sera of BALB/c mice s.c. immunized with 3 different types of rSmeg strains, rSmeg-pMyong2-p24, rSmeg-pAL-p24 and rSmeg-pMV306-p24 and a wild- type strain as a control were analyzed. As shown in Fig. 4d, both rSmeg-pMyong2-p24 and rSmeg-pAL-p24, but not rSmeg-pMV306-p24 elicited significantly higher levels of IgG2a isotype than wild type. With regard to the IgG1 isotype, rSmeg-pMyong2-p24 elicited a lower level of IgG1 than rSmeg-pAL-p24; however, it does not reach statistical significance (P = 0.146). Collectively, the IgG2a/IgG1 ratio, of which a higher level indicates more Th1- biased humoral immune response^32^, was the highest in sera immunized by rSmeg-pMyong2-p24 (1.21) compared to those immunized by other types of Smeg (wild type = 1.05; rSmeg-pAL-p24 = 1.03; rSmeg-pMV306-p24 = 0.97) (Fig. 4d), suggesting that rSmeg-pMyong2-p24 strain can elicit an enhanced HIV-1 p24 Gag-specific Th1-biased humoral response in immunized mice.

### The rSmeg-pMyong2-p24 strain elicits an enhanced HIV-1 p24 Gag-specific cytotoxic T lymphocyte response in immunized mice

**Figure 5:**
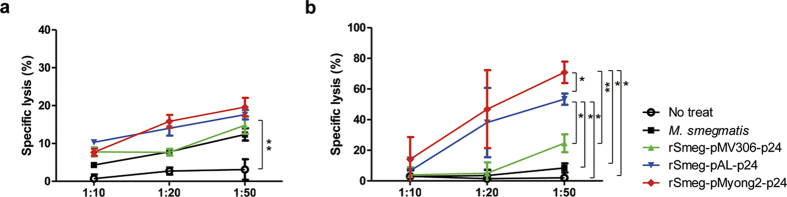
Cytotoxic T lymphocyte responses of mice immunized with recombinant *M. smegmatis* strains. (**a**) CTL responses due to the reaction of *in vitro* stimulated splenocytes with Ag85B and Ag85B transfected P815 cells. (**b**) CTL responses due to the reaction of *in vitro* stimulated splenocytes with p24 and p24 transfected P815 cells. Data are representative of two independent experiments. All the statistical analyses were calculated by comparisons with the values for rSmeg-pMyong2-p24. Means ± SD are shown. *P < 0.05; **P < 0.01; ***P < 0.001.

To test whether rSmeg-pMyong2-p24 elicits an enhanced HIV-1 p24 Gag-specific cytotoxic T lymphocyte (CTL) response in immunized mice, we analyzed CTL activity of splenocytes immunized with 4 different types of Smeg strains, 3 rSmeg, rSmeg-pMyong2-p24, rSmeg-pAL-p24 and rSmeg-pMV306-p24 and the wild- type strain using a LDH cytotoxicity assay. The immunized procedure is described in Fig. 4a. The P815 cells (H-2^d^) transfected with the plasmids harboring p24 or Ag85B-ESAT-6 fusion genes (pcDNA3.3-p24 or pcDNA3.3-Ag85B-ESAT-6) served as target cells and the effector/target ratios were 10:1, 20:1, and 50:1, respectively. The expression of each transfected P815 cell was confirmed by western blot analysis (Supplementary Fig. S4). As shown in Fig. 5, at the E:T ratio of 50:1, the CTLs from mice immunized with rSmeg-pMyong2-p24 could elicit a significant higher level of HIV-1 p24 Gag-specific target cell lysis, compared to those of the other Smeg strains (Fig. 5b). It may be due to the fact that rSmeg-pMyong2-p24 could present the largest amounts of expressed p24 into antigen presenting cells (APCs) among 4 different types of Smeg strains. However, no significant difference in Ag85B specific CTL killing among the 4 strains was found (Fig. 5a). The reason is because almost the same level of Ag85B could be presented into APCs irrespective of vector types or Smeg strains. Indeed, Ag85B orthologue (diacylglycerol acyltransferase/mycolyltransferase, *fbpB*) (GenBank Accession No., NC_008596) showing amino acid sequence homology of more than 70% with *M. tuberculosis* Ag85B was also found in the genome of *M. smegmatis* strain 700084/mc^2^ 155 (GenBank Accession No. NC_008596). So, similar levels of Ag85B-specific CTL response found between mice vaccinated with Smeg strains (one wild type and 3 recombinant strains) may be due to the presence of *M. smegmatis* Ag85B orthologue, of which similar amounts may be expressed between strains. Also, to further confirm and compare the p24 specific CTL responses by rSmeg strains, p24 peptide A9I was also used for the cell cytotoxicity assay. This peptide is known as a major histocompatibility complex (MHC) class I-restricted p24 epitope, especially in BALB/c mouse (H-2^d^)^34^. The result showed that rSmeg-pMyong2-p24 strain could also induce a stronger CTL response (more than 50% of cytotoxicity), compared to those of other rSmeg strains, against P815 target cells pulsed with the A9I peptide (Supplementary Fig. S5). Our data indicated that rSmeg-pMyong2-p24 strain can elicit an enhanced HIV-1 p24 Gag-specific CTL response in immunized mice.

## Discussion

For vaccine development against diseases such as AIDS and tuberculosis, attention has focused on developing strategies for the vaccine induction of cellular immunity, particularly CTL^1^. Studies of laboratory animals and early-phase clinical trials with humans have shown that live recombinant vectors can generate CD4 and CD8 T-lymphocyte responses to a variety of pathogenic microorganisms^35^,^36^,^37^,^38^,^39^,^40^. Of note, the most-effective strategies for the elicitation of cellular immune responses are heterologous prime/boost regimens. Recently, the rSmeg strain of live recombinant vectors has been shown to be useful as priming vectors in prime/boost vaccination regimens for the induction of cellular immune responses against HIV-1 infection by potentiating a vigorous secondary immune response following boosting, particularly expanding a large pool of competent CTLs. It is mainly due to the capacity of Smeg to induce differentiation into antigen specific memory CD8 T cells^41^. However, there is a potential limitation of rSmeg vector that induces a small number of antigen-specific CD8 T cells in comparison to those elicited by other vectors. This limitation may be in part a consequence of the *in vivo* expression of only small amounts of antigen by rSmeg or in part due to the limited access of antigen to the cytosol of infected phagocytes, preventing an efficient MHC class I presentation^41^. Thus, to improve efficacy of rSmeg as priming vectors in prime/boost vaccination regimens, a proper *Mycobacterium* vector system that is particularly efficient in directing transgene products into MHC class I processing pathways should be selected. The simplest approach for this purpose ensures that rSmeg can maintain robust levels of transgene expression, which, mainly depends on the nature of the used vector, such as its copy number and expression capacity of transgene at the transcriptional or translational level.

Therefore, to search a proper *Mycobacterium* vector system facilitating the vaccine efficacy of rSmeg, we compared the HIV-1 p24 expression levels obtained with rSmeg strains using three different vector systems (two episomal vectors, pAL5000 derived vector with 2–6 copies per cell and pMyong2 derived vectors with copy numbers approximately 37 times higher than pAL5000 vector and one integrating vector, pMV306) under the control of a mycobacterial *hsp65* promoter. Our results demonstrate that the best expression was achieved using rSmeg-pMyong2-p24 with the pMyong2 vector (Fig. 2a and b). Also, the rSmeg-pMyong2-p24 stably expressed p24 antigen even after 12 passages of this strain (Supplementary Fig. S2). Furthermore, the more pronounced difference in p24 expression was found in infected phagocytes (Fig. 2c), providing a mechanistic basis regarding the enhanced p24 specific T cell proliferation of BMDCs (Fig. 3b and c), T cell effector function (Fig. 4b and c), particularly in CTLs (Fig. 5 and Supplementary Fig. S5), and Th1- biased humoral immune response (Fig. 4d) of rSmeg-pMyong2-p24.

Comparison of the growth rate of 3 rSmeg strains in 7H9 broth showed that there was growth retardation during the interval between 0 and 48 hrs for the rSmeg-pMyong2-p24 strain compared to the other rSmeg strains (Supplementary Fig. S1). This difference may be attributed into the pressure of maintaining a high copy number of the pMyong2 vector system. This result is consistent with the previous report that BCG or Smeg strains transfected with the pMyong2 derived vector were much slower in colony formation in 7H10 agar than those transfected with the pAL5000 derived vector^28^. This finding hints that rSmeg-pMyong2-p24 may be more attenuated in macrophages or *in vivo* mice infection than other rSmeg strains. Actually, we found that after infection of macrophages, rSmeg-pMyong2-p24 formed colony forming units (CFUs) 2–3 times less than those of rSmeg-pAL-p24 strain or rSmeg-pMV306-p24 strain (data now shown). Given the previous finding that attenuated Smeg elicits stronger immune responses than wild-type strain by presenting more antigens to phagocytes^42^,^43^, it can provide rSmeg-pMyong2-p24 an additive advantage in its vaccine application.

In the current study, we have demonstrated that rSmeg-pMyong2-p24 with pMyong2 shuttle vector system elicited higher levels of HIV-1 p24 Gag protein expression and can deliver more p24 antigens into phagocytes, compared to other rSmeg strains using pAL5000 or pMV306 derived system. We also showed that the strain could enhance T cell proliferation capacity of infected BMDCs and elicit improved T cell effector function and Th1 biased humoral immune response in vaccinated mice. These findings suggest that rSmeg-pMyong2-p24 may be an effective candidate vaccine for HIV-1 or co-infection with both HIV-1 and tuberculosis.

## Methods

### Mice and immunization procedures

Female BALB/c mice (~25 g, 7 weeks old) were purchased from Orient-Bio (Seoul, Korea) and were used for experiments at 8 weeks of age. Mice were subcutaneously immunized with wild type and recombinant *M. smegmatis* strains (rSmeg-pMV306-p24, rSmeg-pAL-p24, and rSmeg-pMyong2-p24) twice at 2-week intervals at the bottom of tail. Two weeks after the final immunization, mice were euthanized by CO_2_ inhalation and their spleens were removed and used for immunological assays.

### Ethics Statement

All animal experiments were performed in accordance with institutional guidelines and the protocol approved by the Institutional Animal Care and Use Committee (IACUC; approval No. of SNU-160118-2-1) of the Institute of Laboratory Animal Resources at Seoul National University.

### Construction of *Mycobacterium*-*E. coli* shuttle vectors for the expression of HIV-1 Gag p24 antigen

To generate *Mycobacterium*-*E. coli* shuttle vectors expressing of HIV-1 Gag p24 antigen (p24 Gag), the heat shock protein 65 gene (*hsp65*) promoter region and DNA sequences encoding p24 antigen were amplified by overlapping PCR of *M. bovis* BCG genomic DNA and pNL4-3-deltaE-EGFP vector (NIH AIDS Reagent Program, Germantown, MD, USA)^44^, respectively. The forward primer sequence for *hsp*65 promoter of *M. bovis* BCG was 5′-TTGGTACCGGTGACCACAACGACGCGC-3′ (*Kpn*I). The reverse primer sequence was 5′-CTGCACTATAGGCATTGCGAAGTGATTCCT-3′. The forward primer sequence for p24 gene was 5′-AGGAATCACTTCGCAATGCCTATAGTGCAG-3′. The reverse primer sequence was 5′-AATCTAGACTACAAAACTCTTGCCTTATGGCCAGG-3′(*Xba*I). Two PCR products were conjugated by overlapping PCR using the *hsp65* promoter forward primer and p24 gene reverse primer. The overlapping sequence (phsp-p24) was digested with *Kpn*I and *Xab*I (NEB, Ipswich, MA, USA) and cloned into *Mycobacterium*-*E. coli* shuttle vector pMV306^29^ with T4 ligase (TaKaRa, Kyoto, Japan). The pMV306-p24 construct was also used as a template for amplification of phsp-p24 to clone into pAL-TOPO and pMyong2-TOPO vectors^28^. The sequence of phsp-p24 was amplified again using forward primer 5′-TTGATATCGGTGACCACAACGACGCGC-3′ (*EcoR*V) and p24 gene reverse primer from the pMV306-p24. PCR product was also digested with *EcoR*V and *Xba*I, and cloned into the plasmids, pAL- and pMyong2-TOPO.

### Production of recombinant Ag85B and p24 proteins from *E. coli*

Recombinant Ag85B and p24 proteins were purified from *E. coli* as previously described^45^ with minor modification. For the expression and purification of fusion protein, *E. coli* BL21 strains (RBC Bioscience, Taipei City, Taiwan) were transformed with pET23a-Ag85B or -p24. Protein expression was induced by adding 0.4 mM isopropyl β-D-thiogalactoside (IPTG, Duchefa Biochemie, Haarlem, Netherlands). Bacterial cells were harvested and disrupted by sonication on ice for 10 min. Sonicated lysates were centrifuged at 1600 ×g for 20 min at 4 °C, and the pellets containing Ag85B and p24 proteins were resuspended in binding buffer containing 4 M urea (Sigma Aldrich, St. Louis, MO, USA). The proteins were purified using Ni-NTA His binding resin (Merck, Darmstadt, Germany), and eluted with elution buffer (300 mM NaCl, 50 mM sodium phosphate buffer, 250 mM imidazole) containing 4 M urea. Purified proteins were dialyzed serially against the elution buffer to remove imidazole, urea and residual salts.

### Generation of rSmeg strains expressing HIV-1 p24 Gag

To generate three different types of rSmeg strains expressing HIV-1 p24 Gag, rSmeg with pMyong2-p24 plasmid (designated as rSmeg-pMyong2-p24), rSmeg with pAL-p24 plasmid (rSmeg-pAL-p24), and rSmeg with pMV306-p24 plasmid (rSmeg-pMV306-p24), each plasmid was electroporated into competent *M. smegmatis* mc^2^ 155 strain using the Gene Pulser II electroporation apparatus (Bio-Rad, Hercules, CA, USA)^46^. Transformants were selected on Middlebrook 7H10 medium (Difco Laboratories, Detroit, MI, USA) supplemented with OADC containing 100 μg/ml of kanamycin. Typically, the selected colonies of transformants from the plates were transferred into 7H9 broth (Difco Laboratories) supplemented with 0.5% glycerol, 0.05% Tween-80, 10% ADC and kanamycin were cultured for 3 days. To compare the growth rate among wild type *M. smegmatis* and rSmeg strains, all the strains were adjusted into 0.2 optical density (OD) at 600 nm, and started the growth curve experiment by measuring OD_600_ values at each time point.

### Determination of the p24 Gag expression levels in rSmeg strains

To determine the p24 Gag expression levels of the rSmeg strains, we performed Western blot and enzyme-linked immunosorbent assay (ELISA) analysis. The pellets of cultured recombinant *M. smegmatis* were suspended in B-PER buffer (Thermo Scientific, Rockford, IL, USA) supplemented with lysozyme (100 μg/ml), DNase (5 U/ml), and proteinase inhibitor. The suspensions were sonicated for 5 min (pulse: 0.3 sec, stop: 0.7 sec) on ice and centrifuged at 13,000 rpm, 4 °C for 15 min. Aqueous phage protein samples were quantified by Quick Start™ Bradford Protein Assay (Bio-Rad, USA). Protein samples from recombinant *M. smegmatis* strains were mixed with sample buffer and boiled at 100 °C for 5 min. The samples were resolved by 12.5% SDS-PAGE gels and transferred to nitrocellulose (NC) membranes. The membranes were blocked with 5% skim milk in TBST for 1 hr at room temperature. Mouse anti-p24 monoclonal antibody (Abcam, Cambridge, USA; 1:1,000 dilution) was added and incubated overnight at 4 °C. After incubation, the membranes were treated with the HRP-conjugated goat anti-mouse secondary antibody (Abcam, 1:2,000 dilution) for 1 hr at room temperature. After each step, the membrane was washed with TBST (0.05% Tween-20). The immune-reactive signals were detected using a WEST-one™ Western blot Detection System (iNtRON, Kyungkido, Republic of Korea) with LAS-3000 (Fujifilm, Tokyo, Japan). As an internal control, mycobacterial Hsp65 (Abcam, 1:1,000 dilution) was used to confirm that the protein concentrations were equal in all samples. To check the stable expression of p24, the p24 expression level from the rSmeg-pMyong2-p24 strain of various passage point (after 1, 4, 6, 8, 10 and 12 passages), was also determined. The passage process was conducted from plate to plate (7H10 agar plate with or without kanamycin) and the colonies from each passage were cultured in 7H9 broth medium for 3 days before performing each experiment. The same amount of proteins was used for detection of p24 levels with the p24 ELISA kit (ABL, Rockville, USA) according the manufacturer’s instructions^47^.

### Generation of bone marrow-derived dendritic cells from mice

Dendritic cells (DCs) were generated from the bone marrow (BM) of 8- to 12-week-old BALB/c mice as previously described^48^. Briefly, the BM cells were flushed out of the femurs and tibias with serum-free Iscove’s modified Eagle medium (IMDM; Gibco Invitrogen, UK). The single cell suspension was filtered through a nylon cell strainer (70 μm Nylon mesh; SPL, Korea), washed twice with complete IMDM supplemented with 10% FBS (Gibco Invitrogen), recombinant mouse GM-CSF (1.5 ng/ml; PeproTech, Rocky Hill, NJ, USA) and mouse IL-4 (1.5 ng/ml; PeproTech, USA), penicillin (100 units/ml; Gibco Invitrogen), streptomycin (100 μg/ml; Gibco Invitrogen), gentamicin (50 μg/ml; Gibco Invitrogen), L-glutamine (2 mM; Gibco Invitrogen), and β-mercaptoethanol (50 nM; Gibco Invitrogen), and seeded at a concentration of 1 × 10^6^ cells per well in a 24-well plate in final volume of 2 ml of complete IMDM. Half of the medium was replaced every other day with an equal volume of complete IMDM for 6 days. The immature BMDCs generated were infected with wild-type Smeg or the three different rSmeg strains expressing p24 or induced to mature by treating the cells with 1 μg/ml lipopolysaccharide (LPS; Sigma Aldrich) for 18 hours.

### Determination of the p24 Gag expression levels in BMDCs and J774.1 cells infected with rSmeg strains

The murine macrophage cell line, J774A.1 cell (American Type Culture Collection, ATCC TIB-67) was maintained at 37 °C and 5% CO_2_ in Dulbecco’s modified Eagle’s medium (DMEM; Thermo Scientific) supplemented with 10% (v/v) fetal bovine serum (FBS), 2 mM glutamine, and essential amino acids. BMDCs were generated from mouse bone marrow as described above. For rSmeg infection, J774A.1 cell and BMDCs were seeded 5~10 × 10^5^cells per well (24-well plate) and cultured for 18 hr. The cells were infected with the three different rSmeg strains at a multiplicity of infection (M.O.I.) of 10. The macrophages were incubated for 2 hr to allow phagocytosis of the bacteria, and the extracellular bacteria were removed by washing with PBS three times. Infected J774.1 cells and BMDCs were incubated for 24 hr. To analysis p24 expression in cells, total proteins of cell pellets were prepared by suspension in RIPA lysis buffer and used to determine of p24 levels using the p24 ELISA kit.

### T cell proliferation assay

To conduct T cell proliferation assay, CD4 and CD8 T cells from p24 protein immunized mice and DCs infected with wild type and rSmeg strains were used. First, mice were injected intravenously with p24 protein. After 7 days, splenocytes were washed with ice-cold FACS buffer [PBS containing 1% bovine serum albumin (BSA) and 1 mM EDTA] and blocked on ice for 30 min with super block solution containing 10% rat sera (Sigma Aldrich), 10% goat sera (Gibco Invitrogen), 10% mouse sera (Sigma Aldrich), and 2.4G2 monoclonal antibody (10 ug/ml; BD Biosciences, San Diego, CA, USA). The cells were subsequently stained with BV421-conjugated anti-CD4 (Clone GK1.5, BD Biosciences) and PE-conjugated anti-CD8a (Clone 53-6.7, eBioscience, San Diego, CA, USA) for 30 min at 4 °C and washed three times with ice-cold FACS buffer. FACS AriaIII instrument (BD Biosciences) was used to sort CD4 and CD8 T cell populations. Immature BMDCs were also infected with the wild-type or the three rSmeg strains (rSmeg pMV306-p24, pAL-p24 and pMyong2-p24) at an M.O.I. of 10 for 24 hours. Proliferation assays were conducted using the fluorescent cytoplasmic tracking dye CFSE (Invitrogen, Carlsbad, USA) as previously described^30^. Sorted CD4 and CD8 T cells were stained with CFSE 5 μM for 4 min at 37 °C and for 4 min in ice. Co-cultured cells were blocked on ice for 30 min with super block solution and stained with CD4 BV421-conjugated anti-CD4 (Clone GK1.5, BD Biosciences) and PE-conjugated anti-CD8a (Clone 53-6.7, eBioscience) for 30 min at 4 °C. The cell cycle profiles were determined using FACS LSRFortessa (BD Biosciences), and analyzed using Flowjo software. All the experiments were conducted in triplicate.

### IL-2 ELISA assay

The amounts of murine IL-2 released in the supernatants of the above co-cultured T cell proliferation assay were also determined by ELISA according to the manufacturer’s instructions (BioLegend, San Diego, CA, USA).

### Enzyme-Linked Immuno Spot (ELISPOT) assay

Splenocytes from mice immunized with wild- type and rSmeg strains were used to conduct ELISPOT assay as previously described^49^. In brief, 96-well PVDF membrane ELISPOT plates were coated with mouse IFN-γ (3 μg/ml, clone AN-18) capture antibody (BD-Biosciences) in PBS and incubated overnight at 4 °C. After discarding the capture antibody, the plates were blocked with 200 μl of RPMI 1640 medium with 10% FBS for 3 hours at 37 °C. After blocking, 5 × 10^5^ splenocytes from vaccinated mice were loaded into each well. For each treatment group, cells were stimulated in triplicate with 5 μg/ml of purified p24 antigen or medium alone in a total volume of 200 μl. The plate was incubated at 37 °C for 24 hours. As a positive control, cells were stimulated with 5 ng/ml of phorbol 12-myristate 13-acetate (PMA) (Sigma-Aldrich) and 500 ng/ml of ionomycin (Sigma-Aldrich). After washing with PBST and PBS (3 times each), biotin-labeled mouse IFN-γ (3 μg/ml, clone XMG1.2) detection antibody (BD-Biosciences) was added to each well and the plates were incubated overnight at 4 °C. The wells were washed again and horseradish peroxidase (HRP)-conjugated streptavidin was added to each well. The HRP reaction was developed with 3-amino-9-ethylcarbazole (AEC) substrate reagent (BD Biosciences). The number of spot forming units (SFUs) per well was counted automatically using an ELISPOT reader (AID ELISPOT Reader, Strassberg, Germany).

### Determination of cytokine production in mice immunized with rSmeg strains

Splenocytes from immunized mice were adjusted to a concentration of 1 × 10^6^ cells/well (96 well microplate, 200 μl volume) in RPMI 1640 medium with 10% FBS and purified p24 protein was added at a concentration of 5 μg/ml for *in vitro* stimulation. The cells were cultured and the supernatants were harvested for determination of IL-2 (BioLegend), TNF-α (eBioscience) (24 hr incubation each), IL-10 (R&D Systems, Minneapolis, MN, USA) and IFN-γ (BioLegend) (72 hr incubation each) using ELISA kits.

### Serum antibody detection

To detect the serum antibody ratio, serum samples were collected from the immunized mice by heart puncture after euthanasia by CO_2_ hyperventilation. Plates (96-well) were coated with purified p24 protein (5 μg/ml) in 0.05 M carbonate-bicarbonate buffer (pH 9.6) overnight at 4 °C. Plates were washed three times with PBST and PBS, and blocked at room temperature (RT) for 1 hour with 5% bovine serum albumin (BSA, in PBST). Serum samples were diluted at a ratio of 1:10 in PBS and 100 μl was added to each well. Plates were incubated for 2 hours at RT, washed three times with PBST and PBS, and incubated for 1 hour with mouse IgG2a and IgG1 antibody (BD Biosciences, 1:1,000 dilution). Thereafter, the plates were washed again and incubated with HRP conjugated streptavidin for 30 min at RT, and reacted with BD OptEIA substrate (BD Biosciences) for 10 min before stopping the reaction with 1N H_2_SO_4_. Optical density (OD) was determined by spectrophotometry at 450 nm^50^.

### Cytotoxic T lymphocyte (CTL) assay

Induced CTL responses were determined as previously described^51^ with minor modification. In brief, P815 cells (H-2^d^) were transfected with the plasmid containing p24 or Ag85B-ESAT-6 (pcDNA3.3-p24 or –Ag85B) with lipofectamine 2000 (Invitrogen, Carlsbad, USA). The expression plasmids were generated by cloning p24 (amplified from pNL4-3-deltaE-EGFP vector) or fused Ag85B-ESAT6 (amplified from genomic DNA of *M. tuberculosis* ATCC 27294) sequences into the pcDNA^TM^3.3-TOPO^®^ TA cloning vector (Invitrogen, Carlsbard, USA). The transfected cells were used as target cells (5 × 10^4^ cells) for cytotoxic T lymphocyte (CTL) assay. Non-transfected cells were used as negative controls. To confirm the expression of each protein (p24 and Ag85B-ESAT6), the lysates of each transfected P815 cell were analyzed by western blot. The process of western blot was described above. In the case of Ag85B-ESAT6 detection, rabbit anti-*Mycobacterium tuberculosis* Ag85B antibody (Abcam, 1:1,000 dilution) and HRP-conjugated goat anti-rabbit secondary antibody (Abcam, 1:2,000 dilution) were used. β-actin (Santa Cruz Biotechnology, Texas, USA; 1:1,000 dilution) was used as an internal control to confirm the same amounts of proteins in each lane. Splenocytes (5 × 10^6^ cells/well) from mice immunized with wild type and rSmeg strains were co-cultured with antigens (p24 or Ag85B, 5 μg/ml) at 37 °C in 5% CO_2_ incubator for 6 days and used as effector cells for CTL responses. Also, to further confirm the specific CTL response against HIV-1 gag, splenocytes from mice of each immunized group were pulsed with the major histocompatibility complex (MHC) class I-restricted p24 peptide A9I (AMQMLKETI)^34^ (10 μg/ml; Peptron, Daejeon, South Korea) and incubated for six days with interleukin-2 (IL-2, 30 U/ml; PeproTech, Rocky Hill, USA) at 37 °C in 5% CO_2_ incubator. In this case, target cells were prepared by incubating P815 cells with A9I peptide (10 μg/ml) for 2 hours before the co-culture of effector and target cells. Cell cytotoxicity was evaluated with lactate dehydrogenase (LDH) assay in U bottom 96-well plates according to the manufacturer’s protocol (CytoTox 96 Non-Radioactive Cytotoxicity Assay; Promega, Madison, USA). In brief, effector cells (splenocytes stimulated with antigens) were added to target cells (transfected P815 cells) in triplicate at different effector/target (E/T) ratios (10:1, 20:1 to 50:1) for 6 hours. From the cultured supernatants, released LDH values were measured by spectrophotometry at 490 nm. The percentage of specific cell lysis was calculated with the following formula: [(Experimental - Effector spontaneous - Target spontaneous)/(Target maximum - Target spontaneous)] × 100 (%).

### Statistical analysis

All the data are shown as the mean ± standard deviation. Student’s *t* test was used to compare the differences between groups and the differences were considered statistically significant when the probability values were less than 0.05.

## Additional Information

**How to cite this article:** Kim, B.-J. *et al*. Recombinant *Mycobacterium smegmatis* with a pMyong2 vector expressing Human Immunodeficiency Virus Type I Gag can induce enhanced virus-specific immune responses. *Sci. Rep.* **7**, 44776; doi: 10.1038/srep44776 (2017).

**Publisher's note:** Springer Nature remains neutral with regard to jurisdictional claims in published maps and institutional affiliations.

## Supplementary Material

Supplementary Figures
