# Supplementary material for: Recombinant Mycobacterium smegmatis with a pMyong2 vector expressing Human Immunodeficiency Virus Type I Gag can induce enhanced virus-specific immune responses
Source: Sci Rep. 2017 Mar 16;7:44776. doi: 10.1038/srep44776 (PMC5353558; doi:10.1038/srep44776)
Supplement: Supplementary Figures [file srep44776-s1.doc]

Recombinant *Mycobacterium smegmatis* with a pMyong2 vector expressing Human Immunodeficiency Virus Type I Gag can induce enhanced virus-specific immune responses

Byoung-Jun Kim, Jeong-Ryeol Gong, Ga-Na Kim, Bo-Ram Kim, So-Young Lee, Yoon-Hoh Kook and Bum-Joon Kim

Supplementary Figure S1

Growth curve of p24 recombinant *M. smegmatis* strains in 7H9 broth supplemented with ADC and 100 ug/ml of kanamycin. In the case of wild type *M. smegmatis* culture, kanamycin was excluded from 7H9 broth. To establish the growth curve, culture aliquots were taken at each time point and the OD600 was measured over a period of 5 days.

Supplementary Figure S2


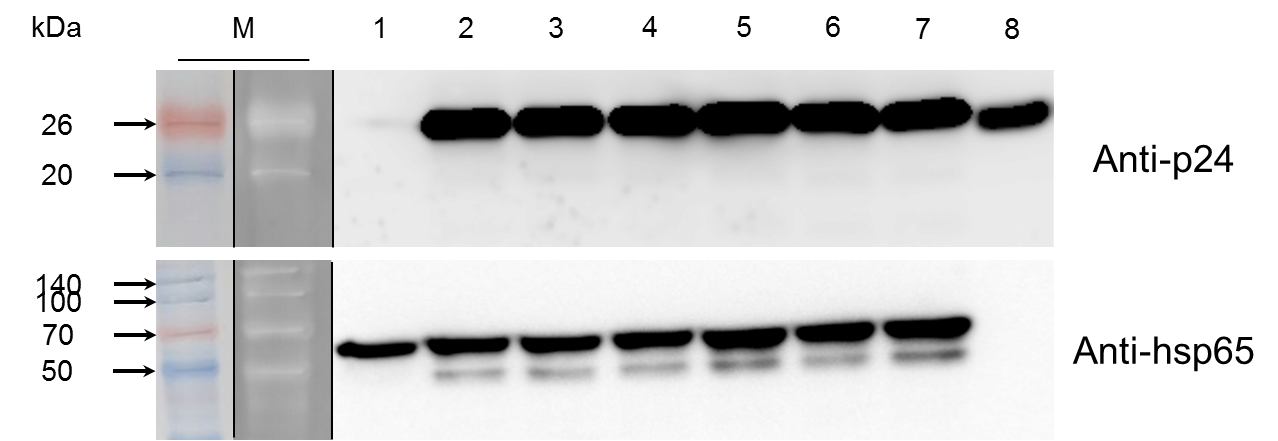


Stability confirmation of p24 expression in rSmeg-pMyong2-p24 strain passaged on 7H10 agar plate with kanamycin by Western blot. Proteins were extracted from wild-type *M. smegmatis* (lane 1) and rSmeg-pMyong2-p24 strain at each passage point (lane 2, first passage; lane 3, after 4^th^ passages; lane 4, after 6^th^ passages; lane 5, after 8^th^ passages; lane 6, after 10^th^ passages; lane 7, after 12^th^ passages). Purified p24 protein was used as a positive control (lane 8). M, molecular weight standard (Elpis Bio, Taejeon, Korea; DokDo-MARK^TM^). Membrane was cropped and probed with Hsp65 antibody (Abcam) as an internal control at the upper size membrane. Distinct membranes were separated by white space. And, marker lane was separated by vertical black line.

Supplementary Figure S3


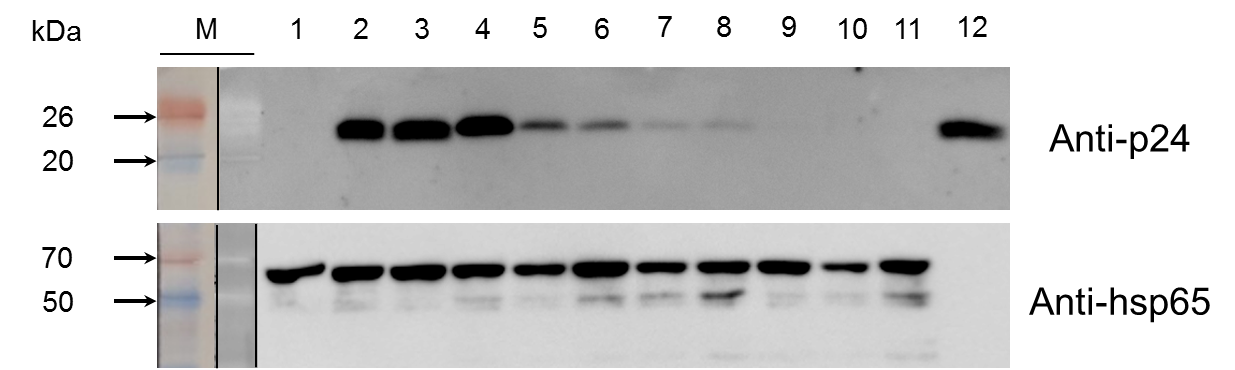


Stability confirmation of p24 expression in rSmeg-pMyong2-p24 strain passaged on 7H10 agar plate without kanamycin by Western blot. Proteins were extracted from wild-type *M. smegmatis* (lane 1) and rSmeg-pMyong2-p24 strain at each passage point (lane 2, first passage; lane 3, after 4^th^ passages; lane 4, after 5^th^ passages; lane 5, after 6^th^ passages; lane 6, after 7^th^ passages; lane 7, after 8^th^ passages; lane 8, after 9^th^ passages; lane 9, after 10^th^ passages; lane 10, after 11^th^ passages; lane 11, after 12^th^ passages). Purified p24 protein was used as a positive control (lane 12). M, molecular weight standard (Elpis Bio, Taejeon, Korea; DokDo-MARK^TM^). Membrane was cropped and probed with Hsp65 antibody (Abcam) as an internal control at the upper size membrane. Distinct membranes were separated by white space. And, marker lane was separated by vertical black line.

Supplementary Figure S4

**
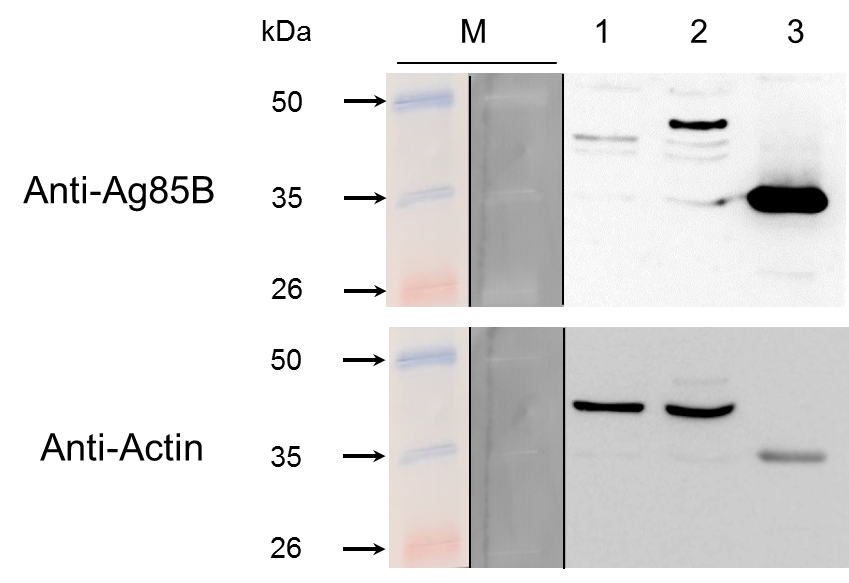
**


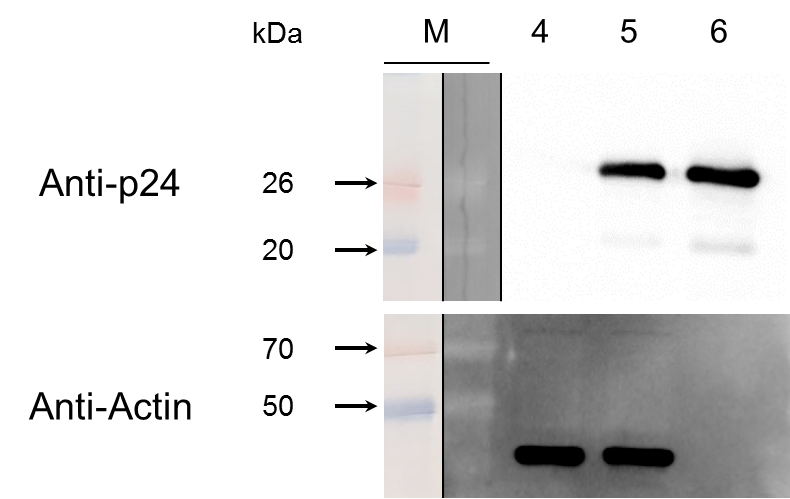


Western blots of pcDNA3.3-p24 and pcDNA3.3-Ag85B-ESAT-6 transfected P815 cell lysates. Lanes 1 and 4, Not-transfected P815 lysates; lane 2, pcDNA3.3-Ag85B-ESAT-6 transfected P815 cell lysates (approximately 43~45 kDa); lane 3, purified Ag85B protein as a positive control; lane 5, pcDNA3.3-p24 transfected P815 cell lysates (approximately 26 kDa); lane 6, purified p24 protein as a positive control; M, molecular weight standard (Elpis Bio, Taejeon, Korea; DokDo-MARK^TM^). After detection of Ag85B expression, membrane was stripped and probed with β-actin antibody (Santa Cruz Biotechnology, Texas, USA) as an internal control. Also, p24 detected membrane was cropped and probed with β-actin antibody as an internal control at the upper size membrane. Distinct membranes were separated by white space. And, marker lane was separated by vertical black line.

Supplementary Figure S5

CTL responses due to the reaction of in vitro stimulated splenocytes with p24 peptide A9I, and A9I pulsed P815 cells. All the statistical analyses were calculated by comparisons with the values for rSmeg-pMyong2-p24. Means ± SD are shown. * *P* < 0.05; ** *P* < 0.01; *** *P* < 0.001.

Supplementary Figure S6


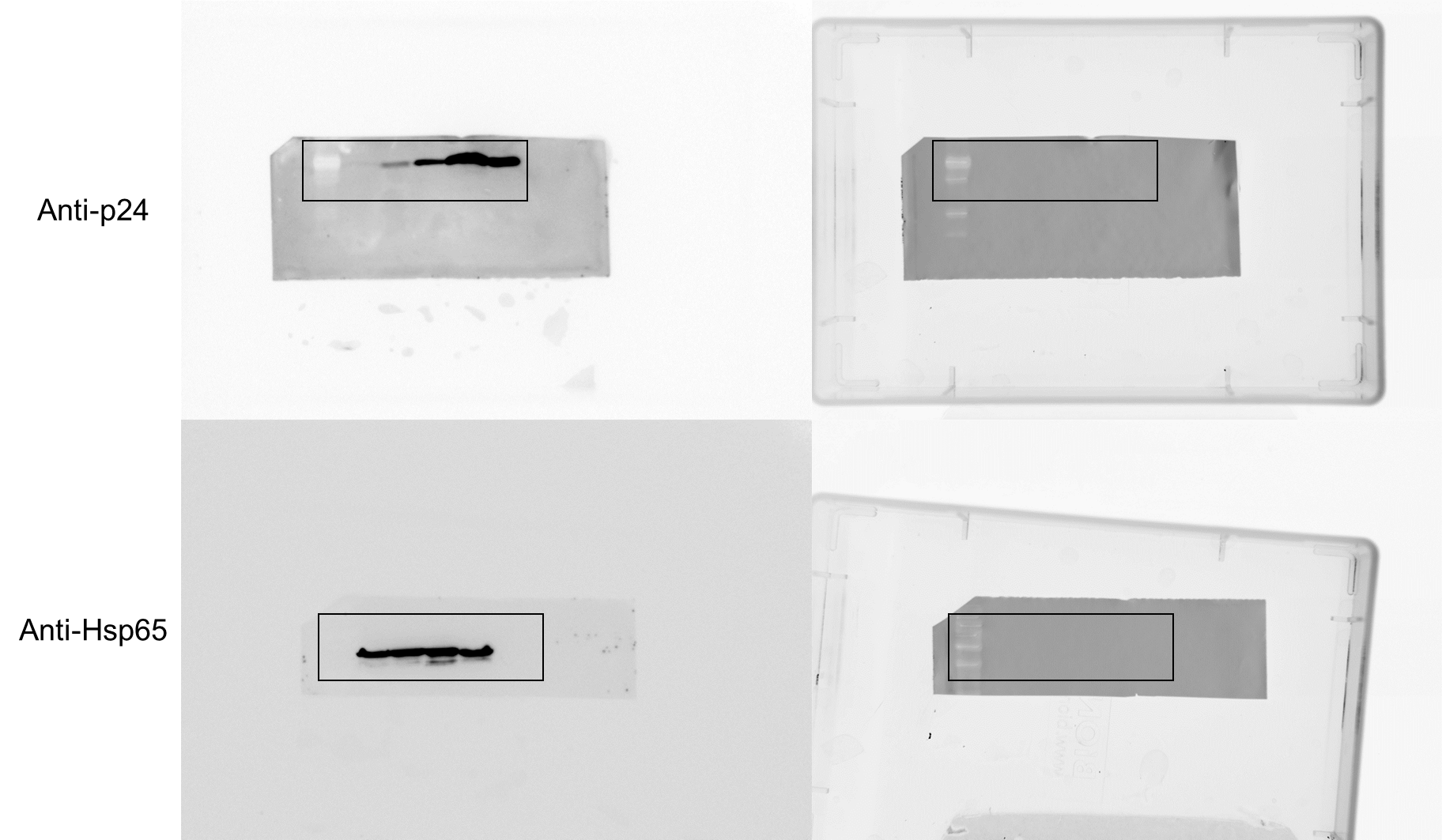


Uncropped, full length original blots of cropped image of Fig. 2b presented in the manuscript. The blots were performed with the indicated antibodies. The cropped area was indicated with the solid lines.

Supplementary Figure S7


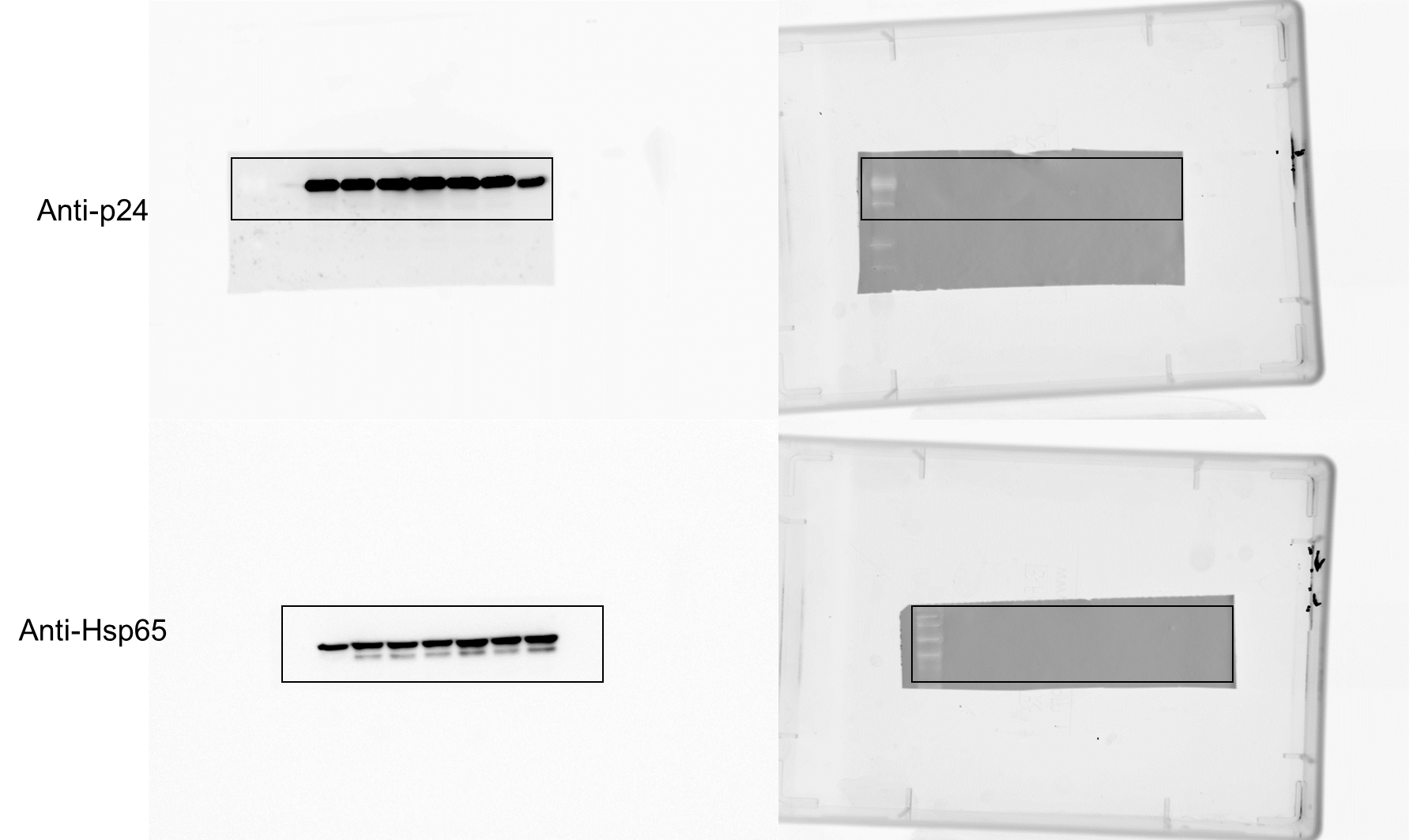


Uncropped, full length original blots of cropped image of Supplementary Figure S2 presented in the Supplementary Information. The blots were performed with the indicated antibodies. The cropped area was indicated with the solid lines.

Supplementary Figure S8


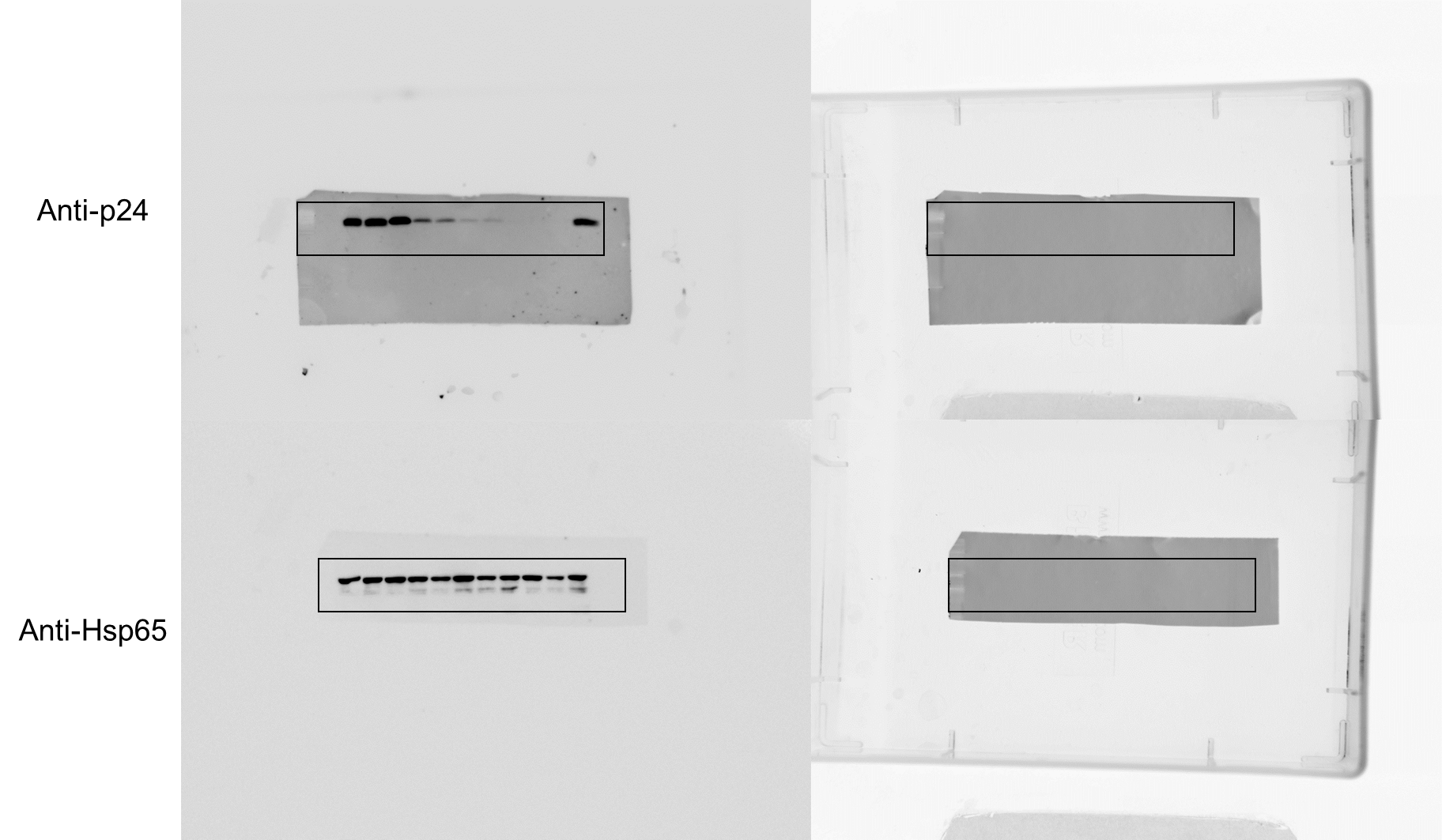


Uncropped, full length original blots of cropped image of Supplementary Figure S3 presented in the Supplementary Information. The blots were performed with the indicated antibodies. The cropped area was indicated with the solid lines.

Supplementary Figure S9


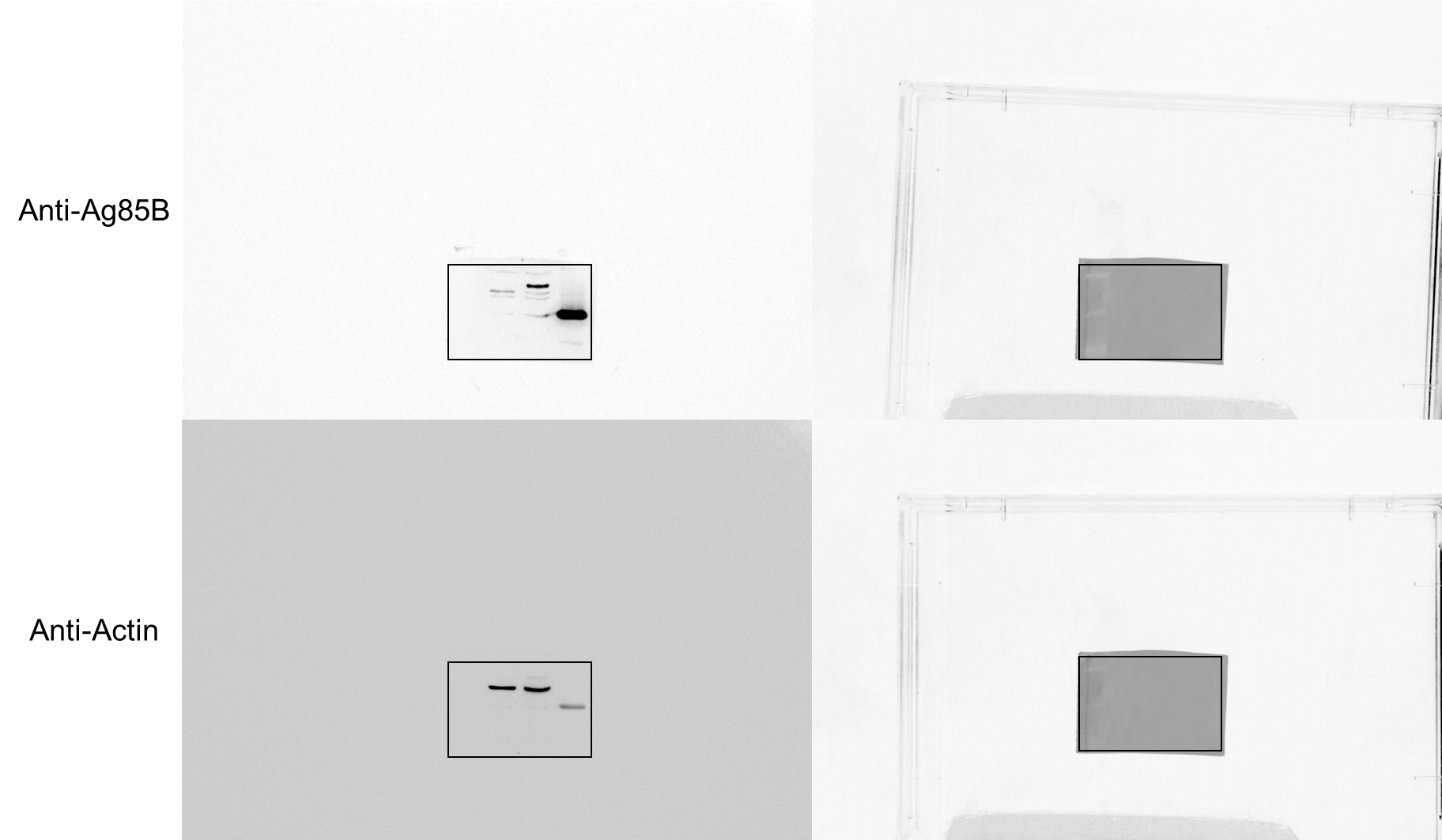


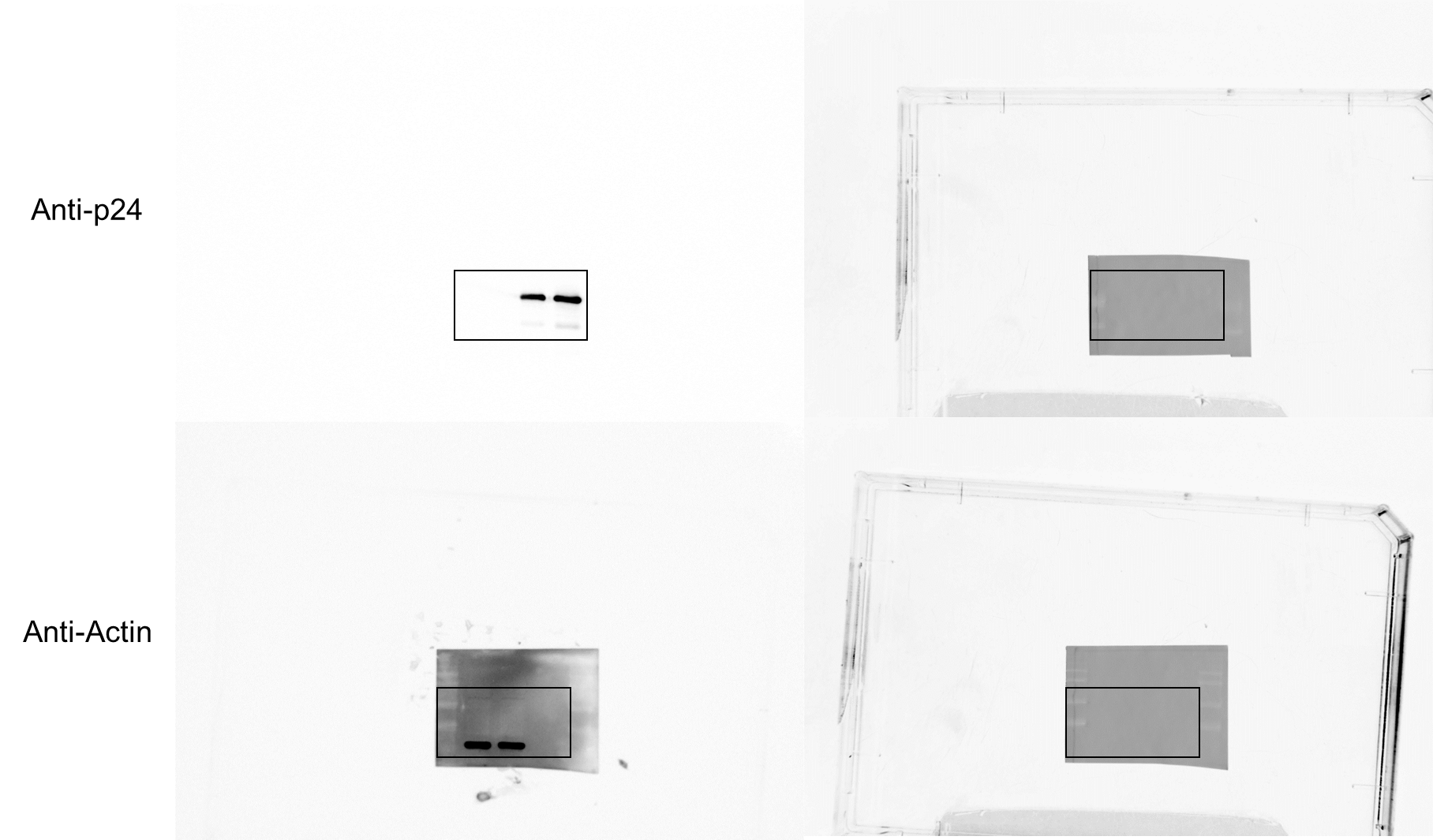


Uncropped, full length original blots of cropped image of Supplementary Figure S4 presented in the Supplementary Information. The blots were performed with the indicated antibodies. The cropped area was indicated with the solid lines.
